# Correlation Between Hearing Metrics and Cognitive Functions in the Older Population in China

**DOI:** 10.1002/alz70860_100724

**Published:** 2025-12-23

**Authors:** Feifan Zhao, Ying Chen, Hao Wu

**Affiliations:** ^1^ Ear Institute, Shanghai Key Laboratory of Translation Medicine on Ear and Nose Disease, Shanghai Jiaotong University School of Medicine, Shanghai, Shanghai, Shanghai, China; ^2^ Shanghai Ninth People's Hospital, Shanghai Jiaotong University School of Medicine, Shanghai, Shanghai, Shanghai, China

## Abstract

**Background:**

The association between hearing loss and cognitive impairment has become a focus of public health research. This study aims to explore the correlations between auditory function at various frequencies and speech recognition thresholds (SRT) against Mini‐Mental State Examination (MMSE) scores.

**Method:**

We analyzed data from the CHOICE‐Cohort study (Chinese Hearing Solution for Improvement of Cognition in Elders), which comprised 17,057 individuals aged 60 and older. Pure tone audiometry was employed to determine hearing thresholds across frequencies from 0.25 kHz to 8 kHz. The SRT was defined as the lowest sound intensity at which participants correctly identified 50% of monosyllabic words. Cognitive function was assessed using the MMSE, and Pearson correlation coefficients were used to evaluate the relationships between hearing metrics and cognitive scores.

**Result:**

Significant negative correlations were observed between auditory thresholds in the better ear and MMSE scores (correlation coefficients between 0.18 and 0.26), particularly stronger at mid and low frequencies than at high frequencies. Furthermore, SRT correlated more strongly with MMSE scores across different cognitive subdomains than pure tone thresholds, indicating that speech recognition may be a more sensitive marker of cognitive health. Additionally, cognitive functions involving orientation, registration, and language were more closely associated with hearing than recall and calculation tasks.

**Conclusion:**

The findings suggest that SRT is more closely correlated with MMSE scores than pure tone thresholds, underscoring the importance of including speech audiometry in routine cognitive assessments for older people.

